# Positive parenting styles and meaning in life in physical education teacher students: a chain mediation of physical activity and smartphone addiction

**DOI:** 10.3389/fpsyg.2026.1811566

**Published:** 2026-04-20

**Authors:** Hui Ma, Tong Liu, Lijun Liu, Zhonggen Yin, Rulan Zhao

**Affiliations:** 1 School of Marxism, Chengdu Sport University, Chengdu, Sichuan, China; 2 School of Sports Training, Chengdu Sport University, Chengdu, Sichuan, China; 3 Sichuan Provincial Sports Museum, Chengdu, Sichuan, China; 4 College of Physical Education and Health Management, Chongqing University of Education, Nanan, Chongqing, China; 5 Engineering University of Joint Logistics Support Force, PLA, Chongqing, China

**Keywords:** meaning in life, physical activity, physical education teacher students, positive parenting styles, smartphone addiction

## Abstract

**Objective:**

This study aims to examine a chain mediation model, investigating whether positive parenting styles influence physical education teacher education students’ meaning in life through sequential mediation of physical activity and smartphone addiction.

**Methods:**

A cross-sectional design was employed, recruiting Chinese physical education teacher education students as participants via stratified sampling. Standardized scales measured positive parenting styles, physical activity, smartphone addiction, and meaning in life. After controlling for demographic variables such as gender and grade level, chain mediation effects were tested using the Bootstrap method.

**Results:**

Positive parenting styles showed a significant positive association with meaning in life (*β* = 0.743, *p* < 0.001). Mediation analysis revealed three pathways through which positive parenting styles indirectly influenced meaning in life: mediated by physical activity (*β* = 0.270, *p* < 0.001); mediated by smartphone addiction (*β* = 0.082, *p* < 0.001); and through a chained mediating effect where physical activity subsequently influences smartphone addiction (*β* = 0.102, *p* < 0.001). The total indirect effect value was 0.454, accounting for 61.104% of the total effect. The Bootstrap confidence intervals for all paths did not include zero, indicating that all mediating effects were statistically significant.

**Conclusion:**

This study demonstrates that positive parenting styles are not only directly associated with a higher meaning in life but may also exert indirect effects by promoting physical activity (independent mediating path), reducing smartphone addiction (independent mediating path), and via the sequential path “physical activity → reduced smartphone addiction” (chain mediating path). The findings provide an integrated perspective for understanding the behavioral mechanisms through which family environments influence the psychological development and professional identity of physical education teacher education students. Future research should employ longitudinal designs to further validate the causal and temporal relationships among these variables.

## 1 Introduction

Globally, college students are facing widespread mental health challenges (Suyuhan et al., 2026). Academic pressures (Ochanda, 2024), societal expectations (Byun and Park, 2014), and the pervasive influence of digital technologies, particularly smartphones (Montagni et al., 2020), interact in complex ways with their psychological adaptation. Physical education teacher education students represent the core workforce for future school sports education, making their physical and mental well-being crucial to their professional development. They must balance intensive specialized athletic training with systematic teacher education curricula. This unique role and its demands may also make them more susceptible to challenges such as professional identity confusion, psychological exhaustion, and fluctuations in meaning in life. Against this backdrop, the importance of meaning in life, an individual’s subjective perception of purpose and value in life has become increasingly prominent (Krok and Telka, 2019). Research indicates that meaning in life is a key predictor of subjective well-being and academic resilience among college students (Li et al., 2021; Cai et al., 2024), while its absence correlates with higher depression risk and maladaptive behaviors (Szcześniak et al., 2022; Jiang and Chen, 2020). Consequently, exploring the mechanisms underlying the influence of meaning in life has become a widely recognized international research topic.

Among various influencing factors, parenting styles, as crucial environmental variables in early development (Yendell et al., 2022), exert enduring effects. They are associated with children’s emotional patterns and behavioral strategies (Gauvain and Perez, 2001; Sarwar, 2016). However, the specific pathways through which this early family factor influences physical education teacher education students’ meaning in life remain incompletely understood. Of particular interest is the potential role of two common behavioral patterns among physical education teacher education students—regular physical activity and excessive smartphone use—in this influence process. Clarifying whether these behavioral variables exhibit sequential mediating effects is crucial for constructing a more comprehensive theoretical model.

Theoretically, positive parenting styles—such as those providing emotional support and autonomy—help satisfy individuals’ fundamental psychological needs (Wei et al., 2022). This fulfillment may translate into intrinsic motivation for managing one’s health, thereby increasing participation in physical activities (Ntoumanis et al., 2018). Engaging in physical activities not only benefits physical health but also helps accumulate psychological resources (Alkhumaish et al., 2024; Zhang, 2020), such as enhanced emotional regulation and self-efficacy (Mu et al., 2024; Yu et al., 2024). According to Conservation of Resources theory (Hobfoll, 1989), these psychological resources form a crucial foundation for effectively managing challenges and preventing maladaptive behaviors (like excessive digital device use). Consequently, individuals with higher levels of physical activity may be better equipped to manage smartphone usage, which may be associated with a lower risk of developing addictive patterns. Extensive empirical research has confirmed that smartphone addiction disrupts individuals’ real-world social functioning, sleep quality (Chu et al., 2023), and goal-directed behaviors (Fabio et al., 2022), all critical dimensions of meaning in life. Consequently, excessive use may directly negatively impact one’s meaning in life.

Based on the above discussion, this study aims to examine a chained mediation model: positive parenting styles may be related to physical activity participation among physical education teacher education students, which in turn may be associated with lower smartphone addiction tendencies, ultimately indirectly linked to higher meaning in life. If this chained mediation model is supported by data, it will align with theoretically derived pathways, providing an integrated analytical framework for understanding potential long-term, indirect mechanisms linking family environments to physical education teacher education students’ mental health. Practically, this research offers theoretical foundations for designing comprehensive professional support programs. By helping candidates transform physical activity participation into constructive psychological resources and effectively manage risk behaviors in the digital age, it strengthens their professional identity and meaning in life.

## 2 Literature review and hypothesis development

### 2.1 Positive parenting styles and meaning in life

Parenting styles serve as a crucial contextual factor in individual development, profoundly influencing psychosocial adaptation. Typically, parenting styles refer to the relatively stable combination of behavioral patterns, emotional atmosphere, and educational philosophies exhibited by parents during childrearing (Baumrind, 1971), forming the primary microenvironment for a child’s growth. In developmental psychology, based on the two dimensions of parental demandingness and responsiveness, researchers typically classify parenting styles into four prototypical categories: authoritative (high demandingness, high responsiveness), authoritarian (high demandingness, low responsiveness), permissive (low demandingness, high responsiveness), and uninvolved/negligent (low demandingness, low responsiveness) (Mccoby, 1983). Among these, the authoritative parenting style, characterized by high expectations and high responsiveness, is considered most conducive to positive individual development, whereas the uninvolved style is generally associated with the most adverse developmental outcomes. Focusing specifically on the core psychological construct of meaning in life, existing research indicates that the family environment—particularly parental parenting behaviors—significantly influences children’s meaning in life. According to self-determination theory, when parents adopt an authoritative and warm parenting style, they effectively satisfy children’s fundamental psychological needs for autonomy, competence, and relatedness (Joussemet et al., 2008). The fulfillment of these intrinsic psychological needs not only forms the foundation of mental health but is also regarded as the driving force behind an individual’s active exploration of the world, integration of life experiences, and construction of personal meaning (Eakman, 2014). Empirical research supports this view, indicating that positive parenting styles are crucial factors in enhancing adolescents’ subjective well-being and life satisfaction (Arslan, 2024). Parental emotional support and the quality of parenting behaviors significantly influence an individual’s development of life purpose and meaning (Kealy et al., 2022). Parenting styles also play a crucial role in children’s cognitive, psychological, and social development (Murry and Lippold, 2018). Parents can further assist children in discovering life’s meaning and purpose by sharing wisdom and values (Lambert et al., 2010). Therefore, we propose Hypothesis H1: Positive parenting styles are positively associated with meaning in life.

### 2.2 Positive parenting styles, physical activity, meaning in life

Parenting styles not only influence children’s psychological adaptation but are also closely linked to their health behavior patterns. This study primarily draws on self-determination theory to explain this relationship. The theory posits that the fulfillment of three fundamental psychological needs—autonomy, competence, and relatedness—serves as a key motivator for individual behavior and development. When these needs remain unmet, motivation and well-being diminish (Ryan and Deci, 2000). Authoritative and warm parenting styles effectively satisfy these needs by respecting children’s choices, setting reasonable expectations, and providing emotional support. This helps children internalize physical activity as a self-motivated, valuable pursuit, thereby positively influencing their activity levels (Hennessy et al., 2010). Controlling or cold parenting environments may hinder the fulfillment of these basic psychological needs. Controlling environments reduce individuals’ intrinsic motivation to exercise by diminishing the satisfaction of these fundamental psychological needs (Matias et al., 2025). Therefore, parenting style is an important predictor of an individual’s physical activity level. Furthermore, participation in physical activities is not only a health-promoting behavior but also a vital pathway for fulfilling basic psychological needs and thereby enhancing meaning in life. Through regular exercise, individuals can experience mastery over their bodies (competence), autonomy in organizing their lives, and potentially build connections in team sports. The fulfillment of these basic psychological needs can transform external behaviors into internal motivation, thereby strengthening university students’ meaning in life (Zhao et al., 2025). Multiple studies also indicate that active participation in physical activity correlates with higher levels of meaning in life (Zhang C. et al., 2025; Zhang Z. et al., 2025). For physical education teacher candidates, physical activity is not only a health behavior but also a core pathway for professional competence development and a vital foundation for occupational identity. The intrinsic motivation fostered by positive parenting styles may lead them to engage more deeply in training, transforming this process into a source of meaning.

In summary, based on self-determination theory, positive parenting styles may influence individuals’ levels of physical activity participation by affecting the fulfillment of their basic psychological needs, ultimately connecting to their meaning in life. Therefore, based on the tenets of self-determination theory, we propose that physical activity serves as a behavioral pathway through which positive parenting styles translate into a greater sense of meaning in life. Hence, Hypothesis H2: Physical activity mediates the relationship between positive parenting styles and meaning in life.

### 2.3 Positive parenting styles, smartphone addiction, and meaning in life

The influence of parental child-rearing styles on individual development is also significantly reflected in behavioral adaptation during the digital age, particularly in smartphone usage patterns. Smartphone addiction refers to a behavioral state where individuals lose control over their smartphone use, resulting in significant negative impacts on their social functioning and mental health (Eduardo et al., 2012). Understanding the familial origins and psychological consequences of smartphone addiction holds significant value for explaining the formation of meaning in life. This study primarily draws upon Self-Determination Theory and Conservation of Resources Theory to explain these associations. On one hand, from the perspective of Self-Determination Theory, controlling or emotionally distant parenting styles may hinder children’s satisfaction of basic psychological needs (Liu et al., 2023), leading them to seek alternative avenues such as smartphones for instant gratification or social compensation (Gugliandolo et al., 2020). On the other hand, authoritative and warm parenting styles promote children’s psychosocial development and self-regulation abilities. From a Conservation of Resources theory perspective, such supportive environments help individuals accumulate psychological resources, whereas controlling or chaotic parenting environments may lead to chronic resource depletion (Hobfoll, 1989). When individuals experience resource depletion, they find it harder to resist the immediate allure of smartphones and lack the self-control resources to effectively manage screen time (Ye et al., 2025). Research supports this, showing that negative parenting styles (e.g., authoritarian and neglectful styles, and the dimensions of rejection and overprotection) directly exacerbate smartphone addiction among adolescents (Lian et al., 2016; Bae, 2015). Smartphone addiction is not merely a behavioral issue but a significant risk factor that erodes one’s sense of life’s meaning. Research confirms that addictive phone use severely disrupts an individual’s practical management abilities, leading to wasted time and reduced efficiency (Wang et al., 2024). This time could otherwise be allocated to goal-oriented activities, deep thinking, and developing high-quality real-world relationships—activities that serve as core sources for constructing one’s life narrative and discovering and experiencing life’s meaning (Wong, 1998). When life becomes fragmented and dominated by passive information consumption, individuals’ perception of purpose and value significantly diminishes. Related studies further indicate a strong negative correlation between smartphone addiction and lower levels of meaning in life (Sun et al., 2026), life satisfaction, and well-being (Hu et al., 2024). Synthesizing the theoretical framework and empirical evidence above, parenting styles may influence children’s psychological needs fulfillment and resource status, thereby correlating with their risk of smartphone addiction. Smartphone addiction, in turn, negatively impacts their meaning in life. Thus, we propose Hypothesis H3: Smartphone addiction mediates the relationship between positive parenting styles and meaning in life.

### 2.4 Positive parenting styles, physical activity, smartphone addiction, and meaning in life

**Figure 1:**
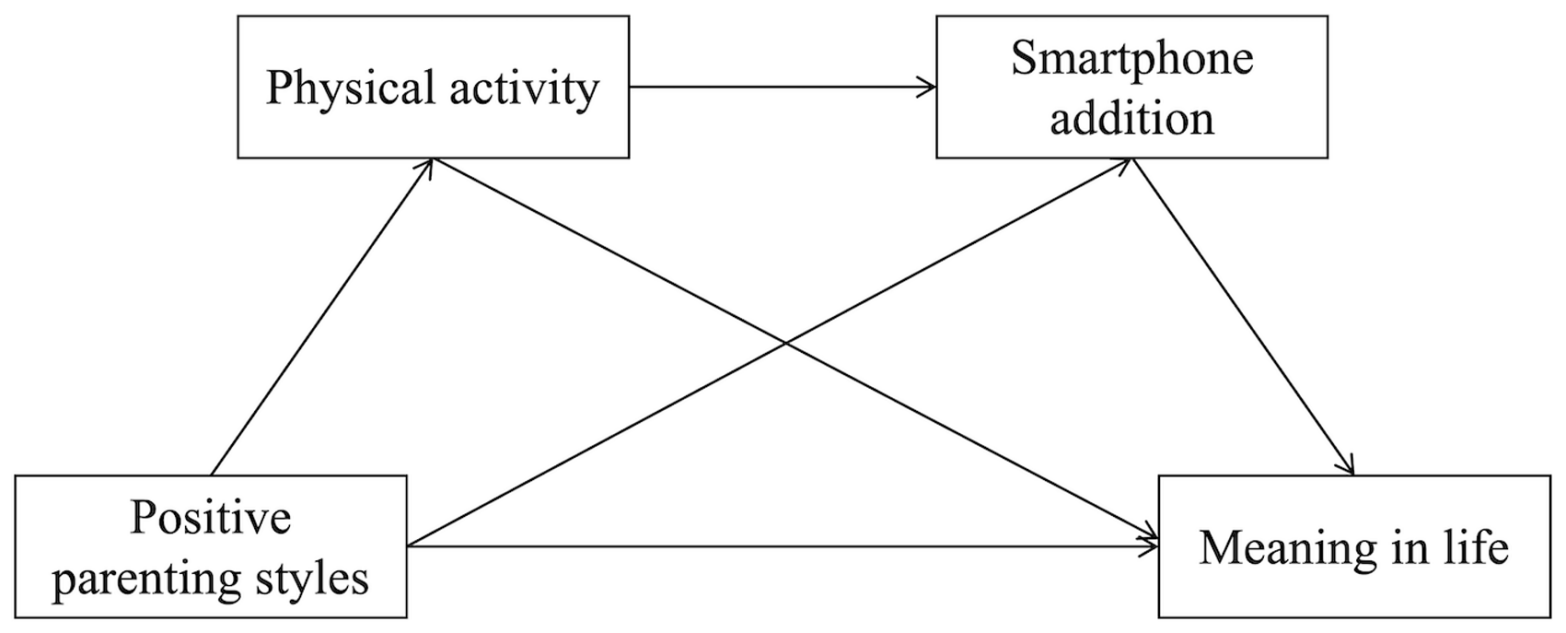
The chain mediation effect model.

Previous analyses have examined how parental child-rearing practices influence meaning in life through both a positive behavioral pathway (physical activity) and a negative risk pathway (smartphone addiction). However, individuals’ positive behaviors and problematic behaviors do not exist in isolation; they often interact and jointly influence the construction of meaning in life. Although previous studies have separately confirmed the positive impact of physical activity on meaning in life (Jiang et al., 2025), and the negative impact of smartphone addiction on meaning in life (Çevik et al., 2020), and an increasing number of studies have incorporated physical activity and smartphone use into the same model (Li et al., 2025), they often examine these variables at different stages of influencing psychological outcomes. Research examining both variables as a mediating sequence is relatively scarce. According to Conservation of Resources theory, a more integrated perspective suggests that psychological resources gained from regular physical activity (such as self-regulation and self-control) can enhance individuals’ self-regulatory efficacy in managing digital behaviors and resisting addiction risks (Wang et al., 2025). Therefore, testing a chained mediation model—“physical activity → reduced smartphone addiction → enhanced meaning in life”(see Figure 1)—rather than a simple parallel mediation model, can validate whether positive health behaviors mitigate the risks of problem behaviors through the dynamic mechanism of building psychological resources. The core purpose of testing this chain model is to validate whether the theoretical pathway from “resource building” to “behavioral regulation” holds. If this associative pattern is established, it will identify potential critical links for future experimental studies or intervention programs aimed at exploring causal mechanisms.

This study proposes that physical activity and smartphone addiction may form a sequential chain of mediation in the process of transmitting family influences to meaning in life. The theoretical foundation for this sequential relationship lies in the dynamic process of psychological resources. As previously noted, positive parenting practices promote physical activity, which in turn helps build psychological resources such as self-regulation and self-efficacy (Wang et al., 2025). These resources, according to Conservation of Resources theory, provide the internal capacity for individuals to manage digital behaviors (Huang et al., 2025). When individuals possess richer psychological resources, they typically regulate smartphone usage more effectively, resulting in a lower risk of addiction (Chen et al., 2025). Conversely, lack of exercise may lead to resource depletion, increasing vulnerability to digital temptations (Huang et al., 2025).

**Figure 2:**
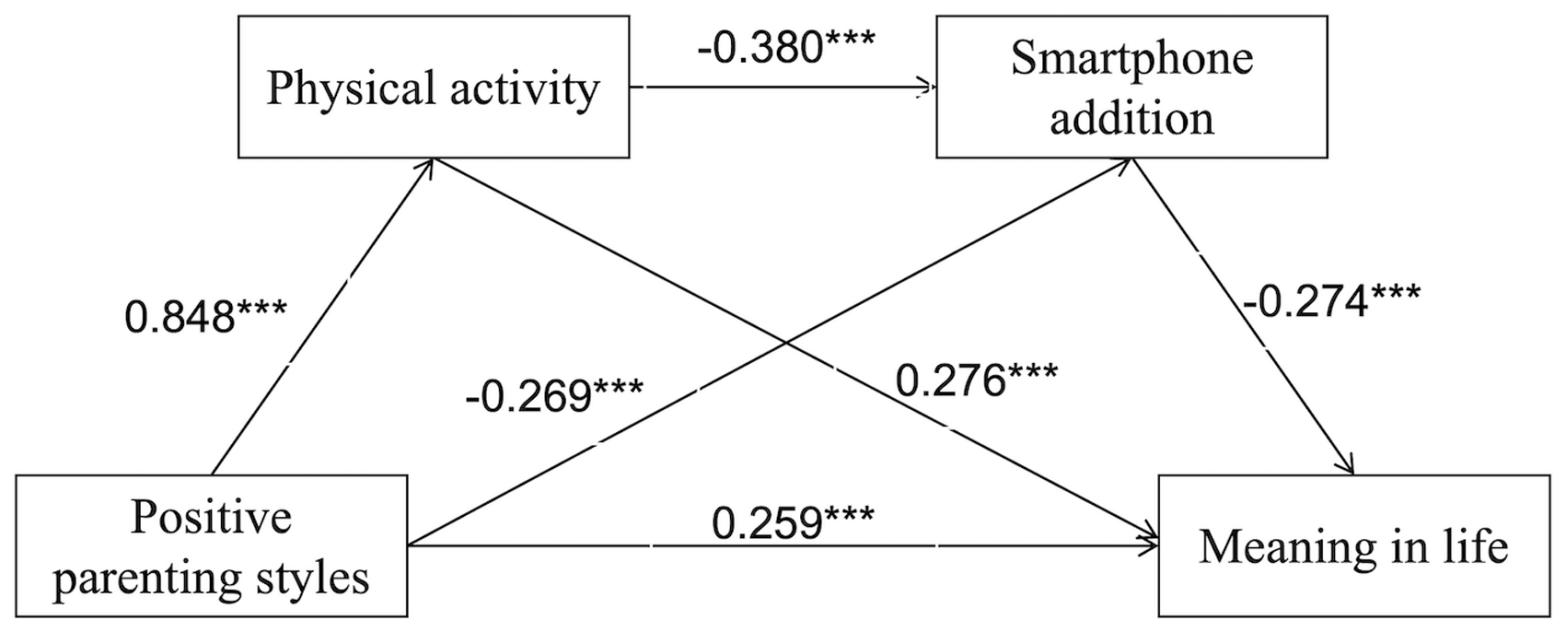
Model of chain mediating roles of physical activity and smartphone addiction between positive parenting styles and meaning in life.

Thus, the core value of testing the chained mediation model—“parenting style → physical activity → smartphone addiction → meaning in life” (see Figure 2)—lies in validating an integrated pathway from family support to professional development. This model posits that a positive family environment helps physical education teacher candidates transform physical activity into stable professional training habits and occupational identity. The psychological resources built through regular training, in turn, enhance their ability to manage digital behaviors, reducing the time and energy consumed by smartphone addiction. Ultimately, this professional lifestyle, grounded in positive training and effective self-regulation, provides robust psychological and behavioral support for their professional commitment to physical education and their meaning in life.

Therefore, we propose Hypothesis H4: Physical activity and smartphone addiction mediate the relationship between positive parenting styles and meaning in life in a chain-like manner.

Positive parenting styles → physical activity → smartphone addiction → meaning in life (Figure 1).

## 3 Methodology

### 3.1 Procedures and participants

This study employed a cross-sectional design, recruiting 1,300 physical education teacher education students from 15 provinces across China’s eastern, central, western, southern, and northern regions through stratified sampling between October 1 and 31, 2025. Questionnaires were administered by physical education teachers. Prior to distribution, the research team provided specialized training to these teachers, covering key aspects of questionnaire design such as voluntary participation procedures, opt-out mechanisms, and confidentiality requirements.

Subsequently, PE teachers distributed the questionnaires to the university students. Participants completed the questionnaires under teacher supervision, during which teachers thoroughly explained the principle of voluntary participation, the meaning of questions, and the right to withdraw. After data collection, 108 invalid responses were excluded based on predefined criteria: (1) Abnormally short or long completion times; (2) Inconsistent response patterns (e.g., repetition or contradictions).

The final sample comprised 1,192 participants aged 18–24 years (mean 19.5, SD 1.265). This included 648 males (54.36%) and 544 females (45.64%). Grade distribution: 471 freshmen (39.51%), 342 sophomores (28.69%), 266 juniors (18.96%), and 113 seniors (9.48%). Household registration type: 580 urban (48.66%), 612 rural (51.34%). The valid response rate was 91.69%. Exclusion criteria included: (1) severe physical illness; (2) history of mental illness; (3) psychological therapy within the past 3 months. Data were collected via an online platform^1^ using an electronic questionnaire comprising five sections: basic information (grade level, gender, height, weight), the Emotional Child-Rearing Practices Scale (EMBU), the Physical Activity Questionnaire (PAQ), the Smartphone Addiction Scale (SAS-SV), and the Meaning in Life Questionnaire (MLQ). Prior to data collection, all participants signed an electronic informed consent form. After data collection, all information underwent anonymization to ensure confidentiality. The anonymized dataset will be securely stored in accordance with institutional ethics guidelines and data management policies, remaining available for research purposes for at least 5 years post-release. Qualified researchers may apply for access to the data, subject to signing a formal data-sharing agreement that safeguards participant privacy and adheres to the original informed consent terms.

### 3.2 Ethics approval and consent to participate

This study was conducted in strict compliance with the ethical principles of the Declaration of Helsinki. The research protocol was approved by the Ethics Committee of Chengdu Sport University (Approval No.: CTYLL2025235). All participants signed an electronic informed consent form via an online platform prior to formal participation. The consent form detailed the study objectives, the principle of voluntary participation, confidentiality agreements, and the right to withdraw from the study at any time without liability. To ensure participant privacy and security, data underwent anonymization during both collection and analysis. The final dataset contained no personally identifiable information.

### 3.3 Measurement tools

#### 3.3.1 Positive parenting style scale

Parenting styles were assessed using Arrindell’s Short-Form Evaluation of Parenting Behaviors (S-EMBU) (Arrindell et al., 1999), revised for Chinese by Jiang et al. (2010). The scale comprises separate versions for fathers and mothers, each containing 21 items organized across three dimensions: Rejection (6 items, e.g., “My parents often get angry with me for reasons I don’t understand”), Emotional Warmth (7 items, e.g., “My parents often praise me”), and Overprotection (8 items, e.g., “I wish my parents wouldn’t worry so much about what I’m doing”). This study used the Emotional Warmth subscale to measure positive parenting style. Items are rated on a four-point Likert scale (1 = Never, 4 = Always), with higher scores indicating greater agreement that the described behavior aligns with how the father or mother treats the respondent. Confirmatory factor analysis was conducted to evaluate the construct validity of the scale. Model fit indices indicated good fit: χ^2^/df = 0.973, GFI = 0.997, AGFI = 0.994, RMSEA = 0.000 (90% CI [0.000, 0.027]). The scale yielded a Cronbach’s alpha coefficient of 0.837 in this study.

#### 3.3.2 Physical activity scale

This study employed the Physical Activity Behavior Scale revised by Liang Deqing to measure participants’ exercise habits over a single month (Deqing, 1994). The scale comprises three factors: physical activity intensity, physical activity duration, and physical activity frequency. Physical activity intensity and frequency were scored on a 5-point scale, while physical activity duration used a 4-point scale. Exercise volume was calculated using the formula “Exercise Volume = Exercise Intensity × Exercise Duration × Exercise Frequency,” with higher scores indicating greater exercise volume per session. The Cronbach’s alpha coefficient for this scale in this study was 0.950.

#### 3.3.3 Smartphone addiction scale

Smartphone addiction was measured using the Smartphone Addiction Scale developed by Kwon et al. (2013). This unidimensional scale comprises 10 items (e.g., “I have delayed planned study or work because of smartphone use,” “I have found it difficult to concentrate on studying or work because of my phone use,” etc.). Each item uses a 6-point rating scale (1 = “Strongly disagree,” 6 = “Strongly agree”), with higher scores indicating greater levels of smartphone addiction. Confirmatory factor analysis was conducted to evaluate the construct validity of the scale. Model fit indices indicated good fit: χ^2^/df = 1.241, GFI = 0.993, AGFI = 0.989, RMSEA = 0.014 (90% CI [0.000, 0.027]). The Cronbach’s alpha coefficient for this scale in this study was 0.923.

#### 3.3.4 Meaning in life scale

Meaning in life was measured using Steger’s the Meaning in Life Questionnaire (MLQ) (Steger et al., 2006), with the Chinese version (C-MLQ) revised by Wang and Dai (2006). This 10-item scale comprises two dimensions: Meaning in Life Experience (5 items, e.g., “I understand my life’s meaning well”) and Meaning in Life Search (5 items, e.g., “I am searching for something that gives my life meaning”). Each item is rated on a seven-point scale (1 = “Strongly disagree,” 7 = “Strongly agree”), with higher scores indicating greater agreement with the statement. Confirmatory factor analysis was conducted to evaluate the construct validity of the scale. Model fit indices indicated excellent fit: χ^2^/df = 0.990, GFI = 0.994, AGFI = 0.991, RMSEA = 0.000 (90% CI [0.000, 0.021]). The Cronbach’s alpha coefficient for this scale in the present study was 0.826.

### 3.4 Data analysis

The data analysis procedure comprised three steps:

1. Common Method Bias Assessment: Harman’s one-factor test was employed to evaluate potential common method bias.
2. Descriptive Statistics and Correlation Analysis: Means, standard deviations, and Pearson correlation coefficients were calculated for all variables (positive parenting styles, physical activity, smartphone addiction, meaning in life).
3. Chain Mediation Analysis: Constructed a chain mediation model using the PROCESS macro (version 4.0) in SPSS 25.0 software. This model examined the mediating effects of physical activity and smartphone addiction to validate the transmission pathway from positive parenting styles to meaning in life. Gender, grade level, and body mass index were included as covariates. Indirect effects were validated using Bootstrap resampling (5,000 iterations) combined with 95% confidence intervals.

## 4 Results

### 4.1 Common method bias test

Since questionnaire data were obtained through self-reporting by participants, the Harman single-factor test was employed to examine common method bias and mitigate its potential impact on research findings. Using SPSS 25.0, exploratory factor analysis was conducted on all items of the Parenting Style Scale, Physical Activity Scale, Smartphone Addiction Scale, and Meaning in Life Scale. Common factors were extracted using principal component analysis, and partial correlations were calculated by isolating the first common factor. Results indicated that the first factor explained 37.330% of variance, falling below the 40% critical threshold. To further assess common method bias, we conducted a confirmatory factor analysis comparing a single-factor model with a multi-factor model (Podsakoff et al., 2003). The multi-factor model exhibited significantly better fit (χ^2^/df = 1.108, CFI = 0.997, RMSEA = 0.010) than the single-factor model (χ^2^/df = 4.312, CFI = 0.782, RMSEA = 0.068), with a significant chi-square difference (Δχ^2^ = 1407.997, Δdf = 30, *p* < 0.001). These results collectively indicate that common method bias does not substantially affect the findings.

### 4.2 Descriptive statistics and correlations

Descriptive statistics and Pearson correlation analyses for the four variables (positive parenting style, physical activity, smartphone addiction, and meaning in life) were conducted using SPSS 25.0 (Table 1. Descriptive statistics and correlation analysis of variables). All variables demonstrated significant interrelationships:

**Table 1:** Descriptive statistics and correlation analysis of variables.

Variable	M	SD	Positive parenting styles	Physical activity	Smartphone addiction	Meaning in life
Positive parenting styles	22.91	1.38	1			
Physical activity	74.93	29.627	0.450**	1		
Smartphone addiction	38.38	10.955	−0.357**	−0.615**	1	
Meaning in life	46.00	9.013	0.391**	0.671**	−0.599**	1

**p < 0.01.

Positive parenting style was positively correlated with meaning in life (r = 0.391, *p* < 0.01); it was positively correlated with physical activity (*r* = 0.450, *p* < 0.01) and negatively correlated with smartphone addiction (*r* = −0.357, *p* < 0.01); Physical activity was negatively correlated with smartphone addiction (*r* = −0.615, *p* < 0.01) and positively correlated with meaning in life (*r* = 0.671, *p* < 0.01); Smartphone addiction was negatively correlated with meaning in life (r = −0.599, *p* < 0.01).

### 4.3 Positive parenting styles and meaning in life among university students: chain-mediated effects

To examine the predictive effects of positive parenting styles, physical activity, and smartphone addiction on meaning in life, this study employed stratified regression analysis. Positive parenting styles, physical activity, and smartphone addiction were analyzed as independent variables, with meaning in life as the dependent variable (Table 2). Results indicated:

**Table 2:** Regression analysis of positive parenting styles, physical activity, smartphone addiction, meaning in life.

Variant	Meaning in life			
	β	T	F	R^2^
Positive parenting styles	0.391	14.657***	214.824	0.152
Physical activity	0.671	31.208***	973.960	0.450
Smartphone addiction	−0.599	−25.834***	667.386	0.359

***p < 0.001.

Positive parenting styles positively predicted meaning in life (*β* = 0.391, t = 14.657, *p* < 0.001); physical activity positively predicted meaning in life (*β* = 0.671, t = 31.208, *p* < 0.001); and smartphone addiction negatively predicted meaning in life (*β* = −0.599, t = −25.834, *p* < 0.001).

This study examined the chain mediating effects of physical activity and smartphone addiction on the relationship between positive parenting styles and meaning in life, with positive parenting styles (X) as the independent variable, meaning in life (Y) as the dependent variable, and physical activity (W1) and smartphone addiction (W2) as mediating variables. The analysis controlled for demographic variables (gender, grade level). Analysis was conducted using the PROCESS macro (version 3.4) in SPSS 25.0 to verify whether positive parenting styles exerted significant mediating and chain mediating effects on meaning in life. Hypothesis testing employed Model 6 of the PROCESS macro, a model specifically designed for testing chain mediation with two mediators in sequential order. Chain mediating effects were validated through 5,000 bootstrap samples and 95% confidence intervals (CIs).

As shown in Table 3. Mediation effect values and effect sizes,positive parenting styles exhibited a significant positive association with university students’ meaning in life (*β* = 0.391, *p* < 0.001; 95% CI [0.339, 0.443]). This indicates that higher positive parenting style scores predict greater perceived meaning in life, validating Hypothesis 1. The relationship between positive parenting styles and meaning in life was mediated by physical activity (*p* < 0.001; 95% CI [0.175, 0.239]), indicating that higher positive parenting styles is associated with meaning in life levels through the mediating pathway of physical activity, thus supporting Hypothesis 2. Smartphone addiction mediated the effect of positive parenting styles on meaning in life (*p* < 0.001; 95% confidence interval [0.014, 0.045]), indicating that positive parenting styles enhance meaning in life by reducing smartphone addiction, thus validating Hypothesis 3. Finally, physical activity and smartphone addiction sequentially mediated the relationship between the two (*p* < 0.001; 95% confidence interval [0.095, 0.092]), demonstrating that positive parenting styles enhance meaning in life through a chained pathway by increasing physical activity and decreasing smartphone addiction, thereby confirming Hypothesis 4.

**Table 3:** Mediation effect values and effect sizes.

Effect pathway	Effect	BOOT SE	BOOT LLCI	BOOT ULCI	Relative mediation effect
Total effect	0.391***	0.027	0.339	0.443	100%
Direct effect	0.083***	0.023	0.038	0.126	21.228%
Total indirect effect	0.308***	0.018	0.275	0.345	78.772%
Indirect effect 1 (physical activity)	0.205***	0.016	0.175	0.239	52.430%
Indirect effect 2 (smartphone addiction)	0.029***	0.008	0.014	0.045	7.417%
Indirect effect 3 (physical activity and smartphone addiction)	0.074***	0.008	0.059	0.092	18.926%

Indirect effect 1: positive parenting styles → physical activity → meaning in life. Indirect effect 2: positive parenting styles→ smartphone addiction → meaning in life. Indirect effect 3: positive parenting styles → physical activity → smartphone addiction → meaning in life. ***p < 0.001.

Furthermore, the effect sizes for all three mediating pathways and the total effect were statistically significant (*p* < 0.001). The total effect size was 0.391, comprising a direct effect of 0.083 (21.228% of the total effect) and an indirect effect of 0.308 (78.772% of the total effect). Among the mediating paths: Path 1 (positive parenting style → physical activity → meaning in life) effect size = 0.205 (52.430% contribution) Path 2 (positive parenting style → smartphone addiction → meaning in life) effect size = 0.029 (contributing 7.417%); Path 3 (positive parenting style → physical activity → smartphone addiction → meaning in life) effect size = 0.074 (contributing 18.926%). In addition, the results of the multicollinearity test showed that all variance inflation factor (VIF) values were less than 5, indicating no multicollinearity issues (see Table 4). Furthermore, the Durbin-Watson (D-W) value was approximately 2, suggesting no autocorrelation in the model, and the sample data exhibited no interdependence. Detailed path diagrams are presented in Figure 2.

**Table 4:** Linear Regression Analysis Results.

Variable	Unstandardized coefficients		Standardized coefficients	t	*p*	Collinearity statistics	
	B	Std. error	Beta			VIF	Tolerance
Constant	32.403	3.555	–	9.115	0.000	–	–
Positive parenting style	0.540	0.149	0.083	3.617	0.000	1.270	0.787
Physical activity	0.139	0.008	0.455	16.823	0.000	1.783	0.561
Smartphone addiction	−0.239	0.0221	−0.290	−11.205	0.000	1.629	0.614
R2	0.512						
*R2*adj	0.510						
F	414.892***						
D-W	2.036						

***p < 0.001.

## 5 Discussion

This study aimed to explore an integrated theoretical model elucidating the complex associations between positive parenting styles and college students’ meaning in life. Using cross-sectional data, the research identifies a significant chain mediating pathway: “positive parenting styles → physical activity → smartphone addiction → meaning in life.” This finding transcends single-mediation perspectives by offering a sequential explanatory framework, revealing serial associations between family environments, individuals’ positive health behaviors, digital risk behaviors, and psychological meaning. It is important to emphasize that, given the cross-sectional design, these findings represent statistical associations rather than causal relationships.

### 5.1 Main findings

The study’s results provide nuanced evidence for understanding the relationship between positive parenting styles and children’s meaning in life. First, the study found that positive parenting styles are significantly associated with meaning in life, aligning with the core tenet of self-determination theory that environments fulfilling basic psychological needs form the foundation for meaning development. This provides new empirical support for applying self-determination theory to early adulthood populations and reaffirms the central argument that the fulfillment of basic psychological needs serves as the deep-seated driving force for constructing meaning in life (Klein, 2019; Vansteenkiste et al., 2020). That is, the extent to which individuals experience meaning in life depends on the degree to which their basic psychological needs are consistently met.

Second, the study separately identified independent mediating effects of physical activity and smartphone addiction, consistent with a statistical mediation framework. The mediating pathway of physical activity aligns with health psychology research, confirming the perspective that physical activity serves as a constructive psychological resource-building activity—a pathway for accumulating positive psychological resources (Li et al., 2024). This indicates that physical activity is far more than mere exercise. Rather, it is a constructive activity that systematically cultivates psychological resources such as self-efficacy and emotional regulation. The mediating pathway for smartphone addiction reinforces numerous findings that excessive use of digital media like smartphones crowds out meaningful life space, thereby diminishing psychological well-being (Stirnberg et al., 2024; Moqbel, 2020). Smartphone addiction may be a resource-depleting factor that disrupts the construction of meaning in life.

However, the study’s most critical advancement lies in its chain mediation model, which reveals that these two mediating variables are not simply parallel but exhibit a significant sequential relationship: “physical activity → smartphone addiction.” This implies that the two mediating effects, previously reported separately in prior research, may actually represent distinct stages of a coherent process. More importantly, this chain model was tested within the specific professional development context of physical education teacher candidates. Findings suggest that autonomous support and emotional warmth derived from the family environment may play particularly crucial motivational and nurturing roles for students who need to internalize high-intensity physical practices as core components of their professional identity. This expands the application boundaries of Self-Determination Theory and Conservation of Resources Theory, demonstrating their robust explanatory power in understanding the early formation of professional identity.

### 5.2 Theoretical contributions and implications

The chained mediating pathway constructed in this study reveals an inherent logical sequence of behavioral substitution mechanisms. This sequence links family environments to psychological outcomes, representing a key theoretical contribution. As suggested by the cross-sectional data, this substitution may represent not merely temporal displacement, but rather a functional transfer and reinvestment of psychological resources and attention. However, longitudinal research is needed to confirm this interpretation. The critical significance of this sequence lies in transcending the common perspective that views physical activity and smartphone addiction as parallel, independent factors. Instead, it clarifies the potential functional connection and sequential order between the two. Specifically, the directional pathway from physical activity to smartphone addiction suggests that the psychological resources and positive experiences gained through regular physical activity may reduce an individual’s need to over-rely on digital devices for emotional regulation or to fill emotional voids. In this way, physical activity may exert a preventive effect. This integrates Self-Determination Theory (explaining motivation from need satisfaction) and Conservation of Resources Theory (explaining how resulting behaviors build resources for subsequent regulation). Self-determination theory explains how positive parenting fosters intrinsic motivation by fulfilling basic psychological needs (Joussemet et al., 2008), while Conservation of Resources theory further elucidates how the resulting positive behaviors accumulate critical psychological resources (Hobfoll, 2001). These resources are subsequently utilized to manage subsequent behavioral choices and mitigate risks. This integrated model offers new insights into a common puzzle in previous research: why individuals’ meaning in life diverges significantly despite similar family backgrounds or stress levels. The study’s deeper theoretical contribution lies in its preliminary revelation of an integrated model of psychological and behavioral mechanisms. This model captures the early socialization process of physical education teachers’ professional identity. This model outlines a latent pathway from “family support” to “professional behavior cultivation,” culminating in “professional psychological resource integration.” It not only explains the long-term impact of parental upbringing but also redefines “physical activity” as a key behavior linking personal habits to professional identity, while identifying “smartphone addiction” as a developmental risk factor that may erode professional time and focus. Consequently, this study offers an integrated perspective emphasizing the development of intrinsic psychological resources in physical education teacher training, addressing the shortcomings of previous research that overly emphasized the transmission of external skills.

### 5.3 Practical significance and application prospects

This cross-sectional study, while unable to establish causality, identifies a chained mediating pathway—“positive parenting styles → physical activity → smartphone addiction → meaning in life”. The observed pattern of associations provides preliminary evidence that can inform future causal investigations.

Future professional training should focus on the potential link between students’ early family environments and their current behavioral patterns. Educators can incorporate supportive parenting principles into teaching—balancing academic demands with emotional support and autonomy cultivation—to help students internalize physical activity as a source of professional identity and meaning. Curriculum design should encourage reflection on the educational value, personal growth, and professional ethos inherent in sports, facilitating the integration of their dual identities as “athletes” and “teachers.”

At the behavioral support level, universities should foster holistic ecosystems that promote physical and mental well-being. On one hand, diverse, structured physical activity opportunities should be integrated naturally into students’ academic and daily lives. On the other hand, building on this study’s findings of a negative correlation between physical activity and smartphone use, health education should guide students to understand the positive effects of regular exercise on energy levels, emotional regulation, and self-control. This foundation can then foster students’ practical abilities to manage digital device usage and establish positive offline social connections.

Furthermore, these findings provide a foundation for dialogue in home-school collaboration. Through appropriate channels, universities can help parents understand the professional development characteristics of physical education majors and the importance of healthy lifestyles, thereby encouraging sustained emotional support. Educators should also address individual differences, assisting students struggling with behavioral adaptation or existential confusion through guided conversations to help them recognize the mutual influence between behavioral habits and psychological states.

In summary, this study supports viewing physical education teacher candidates’ growth through a systemic, interconnected lens—one that simultaneously examines the intrinsic connections between their professional skill development, lifestyle formation, digital behavior management, and pursuit of meaning in life. Building supportive environments grounded in this understanding may positively impact their holistic development. Future research could further test the practical effectiveness of these pathways by designing integrated intervention programs focused on “physical activity promotion” and “digital behavior management.”

### 5.4 Research limitations and future directions

This study has several limitations, which also suggest directions for future research.

First, limitations of the cross-sectional design. While this study preliminarily validated the chained mediation model among variables, it cannot determine causal directionality or temporal sequence. For instance, the negative association between physical activity and smartphone addiction may also be interpreted inversely (i.e., smartphone addiction affecting physical activity). Future longitudinal tracking or experimental intervention studies are needed to further examine temporal relationships and chained mechanisms among variables.

Second, competing theoretical models may exist. Although the proposed model is supported by data, alternative variable sequences (e.g., smartphone addiction as a more proximal mediator) may also hold. Future studies could employ model comparison methods to evaluate the fit of different path models and confirm the optimal explanatory framework.

Third, limited representativeness and generalizability of the sample. The study focused on physical education majors in China, and the applicability of these findings to students from different cultural backgrounds, majors, or educational stages remains to be verified. Subsequent research could examine model stability in broader populations and explore the moderating effects of variables such as gender, personality traits, and social support.

Fourth, self-reported data may introduce common method bias. Future studies could enhance data objectivity and conclusion robustness by incorporating objective measures (e.g., fitness trackers, screen time statistics) or multi-source assessments (e.g., peer and teacher reports).

Nevertheless, the chained mediation model proposed in this study offers a preliminary theoretical framework for understanding the complex interplay among family environment, health behaviors, digital habits, and psychological meaning, providing valuable insights.

## 6 Conclusion

By constructing and testing a chained mediation model using cross-sectional data, this study preliminarily explored the statistical associations linking positive parenting styles to college students’ meaning in life. Findings indicate that positive parenting styles are not only directly associated with meaning in life but may also indirectly relate through a chained pathway involving physical activity and smartphone addiction. Given the cross-sectional design, these findings should be interpreted as demonstrating statistical associations rather than causal sequences. Specifically, data support a model where positive parenting styles first positively correlate with physical activity levels, which then relate to lower smartphone addiction tendencies, ultimately linking to higher meaning in life.

By moving beyond traditional single-mediator approaches, this chained model places positive and negative health behaviors within a dynamic, sequential framework, offering a more integrated mechanistic perspective on the distal effects of family environment. The model suggests that participation in physical activity correlates with lower tendencies toward digital risk behaviors, laying a theoretical foundation for subsequent causal mechanism research.

At the practical level, while cross-sectional data limits causal inference, the revealed association pattern offers valuable insights for college mental health promotion. It supports a systematic intervention logic: cultivating positive lifestyles to prevent problematic behaviors and ultimately foster psychological growth. Encouraging and creating campus environments conducive to physical activity can be regarded as a core health promotion strategy with multiple potential benefits.

Naturally, these findings should be interpreted with consideration of their limitations, primarily the inability of cross-sectional designs to establish causal directionality and potential constraints on sample representativeness. Future research should employ longitudinal designs to validate temporal relationships among variables, test the model’s generalizability across broader populations, and explore additional potential individual or environmental moderators to achieve a more comprehensive understanding of this complex mechanism.

## Data Availability

The original contributions presented in the study are included in the article/Supplementary material, further inquiries can be directed to the corresponding author.

## Footnotes

1
https://www.wenjuan.com/s/UZBZJvYWmq/
